# Research on the variation law of floor stress information entropy in upper protective layer mining based on Brillouin optical fiber sensing

**DOI:** 10.1038/s41598-024-51373-4

**Published:** 2024-01-08

**Authors:** Jing Chai, Gaoyi Yang, Dingding Zhang, Jianfeng Yang, Zhi Li, Zhe Yan, Zixu Wang, Yibo Ouyang, Wulin Lei, Zhongyou Zheng, Kai Sun, Gang Han, Mingyue Weng

**Affiliations:** 1 School of Energy Science and Engineering, Xi’an University of Science and Technology, Xi’an, 710054 Shaanxi China; 2 Key Laboratory of Western Mine Exploitation and Hazard Prevention, Ministry of Education, Xi’an, 710054 Shaanxi China; 3 Shaanxi Coal Huangling Mining Co., Ltd. No. 1 Coal Mine, Yan’an, China; 4 Longdong University Energy College of Engineering, Qingyang, 745000 China; 5 China Coal Energy Research Institute Co., Ltd., Xi’an, 710054 Shaanxi China; 6 Zhongtian Hechuang Energy Co., Ltd., Ordos, 017010 China

**Keywords:** Coal, Fibre optics and optical communications

## Abstract

The characteristics of floor failure and stress changes during the mining process of protective layers are crucial for determining the effectiveness of pressure relief. Three boreholes were designed in the 21104 fully mechanized mining face of Hulusu Coal Mine to implant optical fibers into the floor of the working face. A fiber optic monitoring system was established to monitor the dynamic evolution of stress in the floor rock mass at different mining distances. Based on the information entropy in information theory, the monitoring results in the fiber optic monitoring system are calculated to obtain the stress information entropy at different mining distances. A quantitative dynamic analysis is conducted on the stress change process of the mining floor rock layer, and the stress change law of the protective layer after mining is verified through numerical calculation and similar simulation experiments. The results indicate that the evolution of stress information entropy can be divided into four stages, namely the original rock stress stage, stress concentration stage, stress release stage, and stress recovery stage. The stress information entropy shows a fluctuating upward trend, indicating that coal seam mining leads to a decrease in the orderliness of the overlying rock system and an increase in disorder. In different spatial evolution processes, there are also significant differences in stress information entropy. In the vertical direction, the entropy value of shallow rock layers changes greatly, while the entropy value of deep rock layers changes slightly. Mining leads to a decrease in the orderliness of the entire overlying rock system, an increase in stress information entropy, and a fluctuating upward trend in stress information entropy. The information entropy of overlying rock deformation and re compaction increases, but the degree of change of the former is greater than that of the latter. The Brillouin fiber optic sensing technology provides a new method for monitoring the stress changes in the protective layer mining floor, achieving quantitative analysis of floor rock failure.

## Introduction

When mining coal seams, the priority of mining the upper protective layer is a technical measure to effectively reduce the impact propensity of the protected layer, and it is also the preferred method for mining coal seams with impact propensity^1–6^. After the coal seam mining, the stress state of the roof and floor of the coal seam will change. The redistribution of the stress of the floor of the coal seam will increase the stress of the floor of the coal seam under the coal wall of the working face, resulting in the destruction of the underlying coal and rock mass under the floor^7–9^.

At present, the mature theories of floor failure at home and abroad include water inrush coefficient method^10–12^, rock water stress relationship method, floor “lower three zones” and “lower four zones”^13,14^, in-situ tension crack and zero position failure theory, thin plate model and key layer theory, etc. Based on the theories of modern damage mechanics and fracture mechanics, Shi Longqing^15–17^ combined with the theory of ground pressure, divided the complete rock zone into new damage zone and original damage zone on the basis of the previous “three zones” of the stope, and proposed the “four zones” theory of the stope floor. Mengxiangrui^18^ simplified the stress condition of the coal seam floor under the coal wall as a triangular band load from the coal wall to the peak of the advance abutment pressure and a trapezoidal band load in front of the peak of the advance abutment pressure on the semi-infinite elastomer. According to the Moore Coulomb criterion, the judgment formula of the floor failure was obtained. Liuzenghui^19^ applied a concentrated force P on an isotropic homogeneous semi-infinite plane through elasticity, and a point in the bottom plate (R, θ) Based on the superposition principle, the calculation results of concentrated force are extended to the stress condition of semi-infinite plane body under uniformly distributed load on the free boundary. Gao Zhaoning^20^ believed that the change of mining stress field changed the stress field of coal seam floor, and established the floor calculation mechanical model. Yuan^21^ established the theoretical calculation model of vertical and horizontal shear stress of coal and rock in the floor, and obtained the discrimination criteria of gas conducting fracture zone and pressure relief desorption zone of underlying coal and rock in the upper protective layer. Lei Bai^22^ believed that the vertical stress increase area of deep mining was “bubble like”; The shear stress is generally butterfly shaped; The distribution of plastic zone is “rectangular”, and shear failure is dominant in the periphery and middle. The range of plastic zone decreases with the increase of depth. Xie Guang Xiang^23^ proposed the principal stress transfer coefficient K, and it is considered that the stress shell composed of high stress bundles of the surrounding rock of the floor makes the redistribution of the floor relief stress field selective. Guo Jing Zhong^24^ analyzed the structure and stability of the broken floor rock beam and found that due to the low tensile strength of the rock layer, the floor rock beam cracked first at the lower end of the supports on both sides, and then at the middle and upper part of the beam. Zhang Feng Da^25–27^ established a prediction model of floor failure depth through multi factor coupling and predicted the floor failure depth of North China type coal seam. The results showed that the predicted failure depth of the model was very consistent with the actual failure depth of the working face. Xiao Hong Tian^28^ used the research method of damage mechanics to establish the damage rheological fracture mechanics model of fractured rock mass to analyze the stability of coal seam floor in Zhao ge zhuang mine. Niu Jian Chun et al.^29^ found through the simulation experiment that with the increase of the depth of the coal seam floor, the stress and deformation degree of the coal seam floor decreased. Based on fracture mechanics and renormalization group theory, Li Jia Zhuo^30^ studied the dynamic instability mechanism of damaged rock strata under mining disturbance from the macro and micro perspective. Du KuShi^31^ theoretically analyzed the stress distribution law of the coal and rock mass on the floor, and provided the judgment basis for the gas conducting fracture zone and the pressure relief desorption zone. In summary, there are rich research results on the stress distribution and failure mechanism of the bottom plate, but there is a lack of on-site monitoring of the stress distribution and failure evolution characteristics of the bottom plate, and there is no on-site quantitative analysis process for the stress distribution and failure evolution characteristics of the bottom plate.

In this study, Brillouin fiber optic sensing technology is utilized to monitor the stress information entropy of the floor rock strata during the mining process of the overlying 2–1 coal seam at the coal mine site. The stress evolution behavior of the goaf floor is quantitatively analyzed through a combination of theoretical analysis, numerical simulation, and similar simulation experiments, providing insights into the stress and displacement evolution characteristics of the floor rock strata and the failure mechanism of the floor rock strata. The findings of this study serve as a theoretical basis for ensuring the safe mining of the underlying 2–2 coal seams.

## Calculation method of stress information entropy based on optical fiber monitoring

The concept of information is broad and cannot be fully grasped with a simple definition. However, entropy, as a measure of the uncertainty of a random variable described by a probability distribution, provides a framework for understanding information. In 1948, Shannon proposed the concept of information entropy, which extended the idea of thermodynamic entropy.

The occurrence of a disaster system can be viewed as a process of dimensionality reduction and ordering, which can be described using the evolution of information entropy.

The stress evolution process of the floor after mining the protective layer can also be seen as a transition from disorder to order. To capture the dynamic evolution characteristics of floor stress during the mining process of the upper protective layer, we establish the concept of “stress information entropy” by applying entropy principles.

Stress information entropy comprehensively reflects the dynamic evolution of stress in the overlying rock system as mining progresses.

Stress variation is closely related to the generation, propagation, and closure of fractures. Therefore, analyzing the change pattern of stress information entropy at different mining distances provides valuable insights into the stress variation characteristics of the floor in the mining of the upper protective layer.

In the context of the entire overlying rock system, the stress at each monitoring point can be represented as ${\theta }_{i}$ (i = 1, 2, 3…, n). Then for the entire overburden system, the sum of stresses *U* is

1 $$U = \sum\limits_{i = 1}^{n} {\partial_{i} }$$

enable

2 $$P_{{\text{i}}} = \frac{{\partial_{{\text{i}}} }}{U}\quad \left( {{\text{i}} = 1,\;2,\;3, \ldots ,\;{\text{n}}} \right)$$

So $\sum\nolimits_{i = 1}^{n} {P_{i} = 1}$ (i = 1, 2, 3, …, n). Satisfying the normalization condition, the mechanical interpretation of it is that each monitoring point's stress represents the proportion it holds among all the stress values at the monitoring points. In other words, *P*_*i*_ (*i* = 1, 2, 3, …, n) describes the distribution of stress in the overlying rock, where n represents the number of stress monitoring points.

Then the stress information entropy* H* can be defined as

3 $$H = - \sum\limits_{i = 1}^{n} {P_{i} \log_{2} } P_{i}$$

Based on stress data collected from monitoring points using fiber and fiber grating sensors, it is possible to calculate the entropy of stress information in the overburden system. Through an analysis of the fluctuation in stress information entropy, the evolutionary pattern of stress in the underlying strata is unveiled, along with the relationship between stress and fractures. This facilitates the identification of stress variation patterns in the underlying strata during the excavation of the upper protective layer.

**Figure 1:**
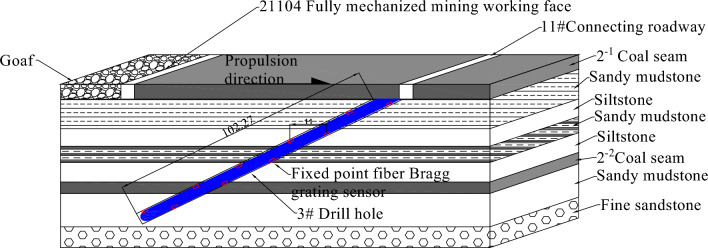
Profile of Borehole 3#

Figure 1 illustrates the longitudinal section of borehole 3#. Distributed fiber optic cables are arranged along the borehole, forming a circular loop at the bottom. Thirty point-type fiber Bragg grating (FBG) sensors are designed, with ten sensors implanted in each borehole at equidistant intervals. The distributed fiber optic sensing technique based on Brillouin optical time-domain analysis (BOTDA) enables extensive real-time monitoring of rock deformation within the range of fiber deployment. The quasi-distributed fiber Bragg grating (FBG) sensing technique facilitates high-precision point-based monitoring of rock deformation within the range of FBG deployment. Stress data is measured and the stress information entropy is calculated using the corresponding formula.

## Field industrial test

### Project overview

The Hulusu Coal Mine is located in the Hujierte Mining Area of the Dongsheng Coalfield. The mine field runs approximately 7.4 km from north to south and spans about 13.0 km in width, with a total area of approximately 92,761 km^2^. The designed recoverable reserves of the mine are 1.624 billion tonnes. The designed production capacity of the mine is 13.0 million tonnes per annum, with an approved production capacity of 8.0 million tonnes per annum. The mine adopts a development method with three main, auxiliary, and ventilation shafts. The first horizontal roadway is located at the 2–2 Medium Coal seam, with an elevation of + 640 m. The coal-bearing formation in this mining area belongs to the Middle Jurassic Yan’an Formation, consisting of five coal seams numbered 2, 3, 4, 5, and 6. There are a total of 8 workable coal seams, with a combined thickness ranging from 22.18 to 36.85 m. The total recoverable reserves of the entire mining area amount to 1692.64 million tones. Among them, the recoverable reserves of the 2–1 coal seam and 2–2 medium coal seam are 466 million tones, with a service life of 25.6 years. The dip of the 2–1 coal seam ranges from − 3° to + 3°, with an average thickness of 2.54 m and a mining height ranging from 2.0 to 2.97 m, averaging at 2.54 m. The 2–2 medium coal seam has an average burial depth of approximately 663 m. The dip of the coal seam ranges from − 3° to + 3°, with an average mining height of 3.85 m.

### Mining conditions of coal seam in working face

The mining face for the 21104 integrated mining is located in the middle of the Hulusu Wellfield 2–1 coal block, and it is the third mining face. The ground elevation ranges from + 1304.2 to + 1326.1, and the roof elevation ranges from + 672 to + 681. The length of the mining face along the strike is 3495 m, with a mining length of 3015 m and a working face length of 320 m. The mining area covers 964,000 square meters. The average burial depth of the mining face is approximately 635 m. The coal seam has a dip angle of 0° to 3°, with an average thickness of 2.63 m, a bulk density of 1.31 t/m^3^, a geological reserve of 3.365 million tons, and a recoverable reserve of 3.2304 million tons. The north part of the working face is served by three main haulage roads for the entire eastern wing.

**Figure 2:**
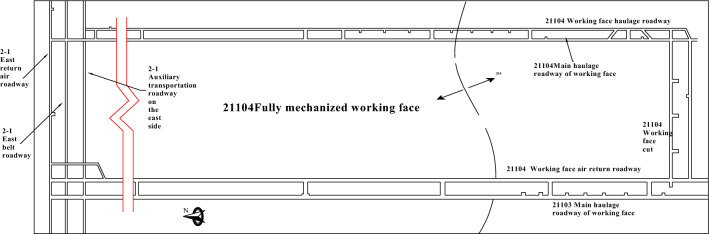
Roadway layout plan.

The 21104 fully mechanized mining face is equipped with four roadways, namely, the auxiliary transport roadway (5.4 × 2.8 m), the transportation roadway (5.4 × 2.7 m), the return airway (5.0 × 2.6 m), and the main cutting eye (7.8 × 3 m). Among them, the return airway of the 21104 face is a leftover roadway from the previous auxiliary transport roadway of the 21103 face. All four roadways are arranged along the roof of the coal seam. Please refer to Fig. 2 for the layout plan of the roadways.

### Fiber optic monitoring system layout

The bottom strata unloading fiber monitoring system is composed of sensing fibers, signal transmission fibers, and signal processing terminals.

Distributed fiber sensing technology, such as BOTDA (Brillouin Optical Time Domain Analysis), enables extensive real-time monitoring of rock deformation within the range of the deployed sensing fibers. On the other hand, quasi-distributed sensing technology using Fiber Bragg Grating (FBG) allows for high-precision point-wise monitoring of rock deformation within the range of the deployed FBG sensors^32^.

**Figure 3:**
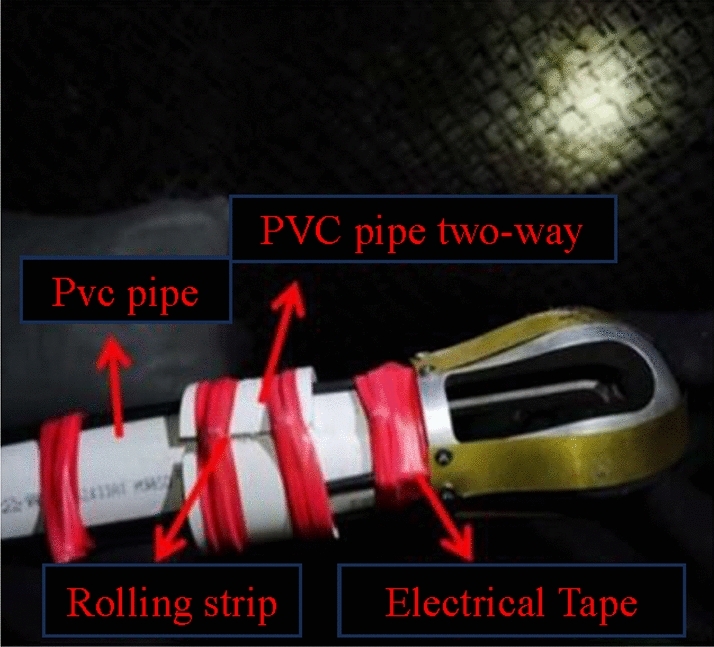
Connection position of sensing optical cable, metal guide and PVC pipe.

The optical fiber sensing monitoring system, as shown in Fig. 3, is connected by the borehole sensing optical cable and the underground signal transmission optical cable. The sensing signal of the borehole sensing optical cable is transmitted to the FBG-BOTDA data monitoring instrument arranged on the surface through the signal transmission optical cable^33^.

**Figure 4:**
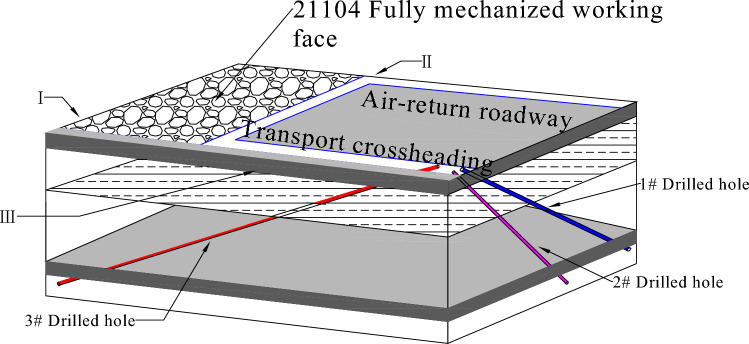
Three dimensional schematic diagram of optical fiber drilling arrangement.

Based on the specific engineering conditions of the HuluSu coal mine and the monitoring objectives of this project, three boreholes were drilled into the floor of the working face in the 11 joint roadway of the 21104-working face. Sensing optical cables were then inserted into these boreholes for monitoring purposes. The FBG-BOTDA technique was utilized to jointly monitor the stress variations in the floor coal and rock mass, aiming to uncover the spatiotemporal relationship of pressure relief during the mining of closely spaced coal seams^34^. The three-dimensional schematic diagram illustrating the layout of the fiber boreholes is depicted in Fig. 4, and the drilling parameters are provided in Table 1.

**Table 1:** Drilling parameters.

Drill hole number	Azimuth (°)	Dip angle (°)	Aperture (mm)	Borehole depth (m)	Vertical height (m)	Dip offset (m)	Strike offset (m)
1#	270	15	110	133	34.42	128.47	0
2#	270	45	110	37	26.16	26.16	0
3#	200	20	110	108	36.94	34.71	95.37

Distributed optical fibers were arranged along the 1st, 2nd, and 3rd boreholes, forming a circular loop at the bottom of each borehole. The actual sensing lengths of the fiber cables inside the three boreholes were 74.0 m, 216.0 m, and 266.0 m, respectively.

The fiber cable sensing on the floor was installed at vertical depths of 26.2 m, 34.4 m, and 36.5 m, respectively. In total, 30 point-type Fiber Bragg Grating (FBG) sensors were deployed, with 10 sensors implanted in each borehole, evenly distributed. Unfortunately, during the installation process, two gratings in the 2nd borehole were damaged, leaving 28 gratings functioning properly for monitoring purposes.

### Stress analysis of floor coal and rock mass

**Figure 5:**
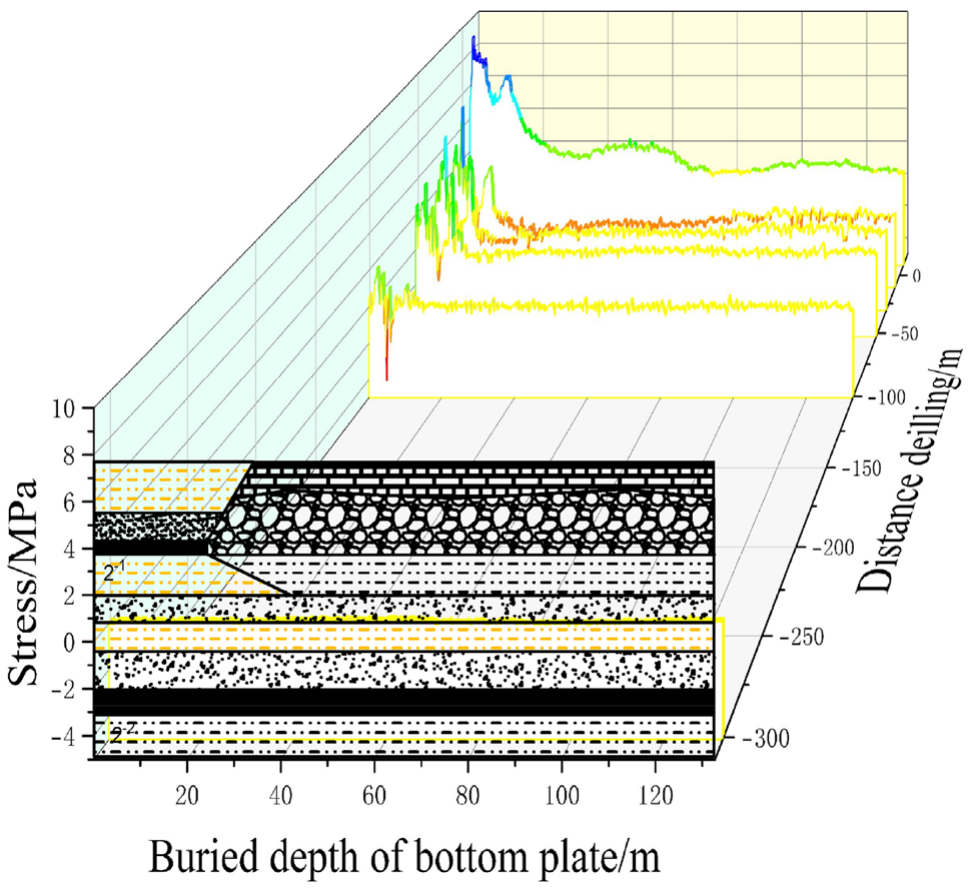
1# Borehole optical fiber monitoring data.

Figure 5 showcases the stress variation curve extracted from the fiber optic cables located in borehole 1#, which were arranged parallel to the strike direction. As depicted in the graph, it is evident that during the progression of the working face, when the working face is situated at a considerable distance from the borehole, the fiber optic cables inside the borehole remain unaffected by the mining activities. This indicates that the influence range of the advancing working face is limited, and the borehole is not yet impacted by mining activities. When the working face is 50 m away from the borehole, the cable below the working face at the borehole entrance is initially affected, leading to stress changes. This change primarily occurs due to the concentrated stress on the floor strata caused by the vertical transmission of pressure from the coal pillar, resulting in the formation of shear stress planes and deformation in the corresponding rock layers. At this stage, the changes occurring in the floor strata below the working face are within the margin of error, suggesting that the working face floor remains unaffected by mining activities. When the working face advances to a distance of 10 m from the borehole, significant stress changes are measured in the cable below the working face inside the borehole. The maximum stress change occurs at a depth of 8.34 m, reaching 1.86 MPa, while the minimum stress change occurs in the strata at a depth of 26.54 m, with only 0.24 MPa. Shallowly buried rock layers experience a greater impact from stress concentration compared to deep-buried rock layers. As the working face surpasses the 10 m mark of the borehole, the floor strata are no longer subject to stress concentration. As the working face advances, the expansion stress in the floor strata is released, leading to a decrease in stress. The greatest magnitude of stress release occurs at a depth of 2.84 m, with a stress change of 6.53 MPa, while the minimum stress release at a particular location corresponds to a rock layer stress release of 0.45 MPa. Subsequently, due to the damage caused by floor expansion, the distributed fiber optic data collection in borehole 1# concludes.

**Figure 6:**
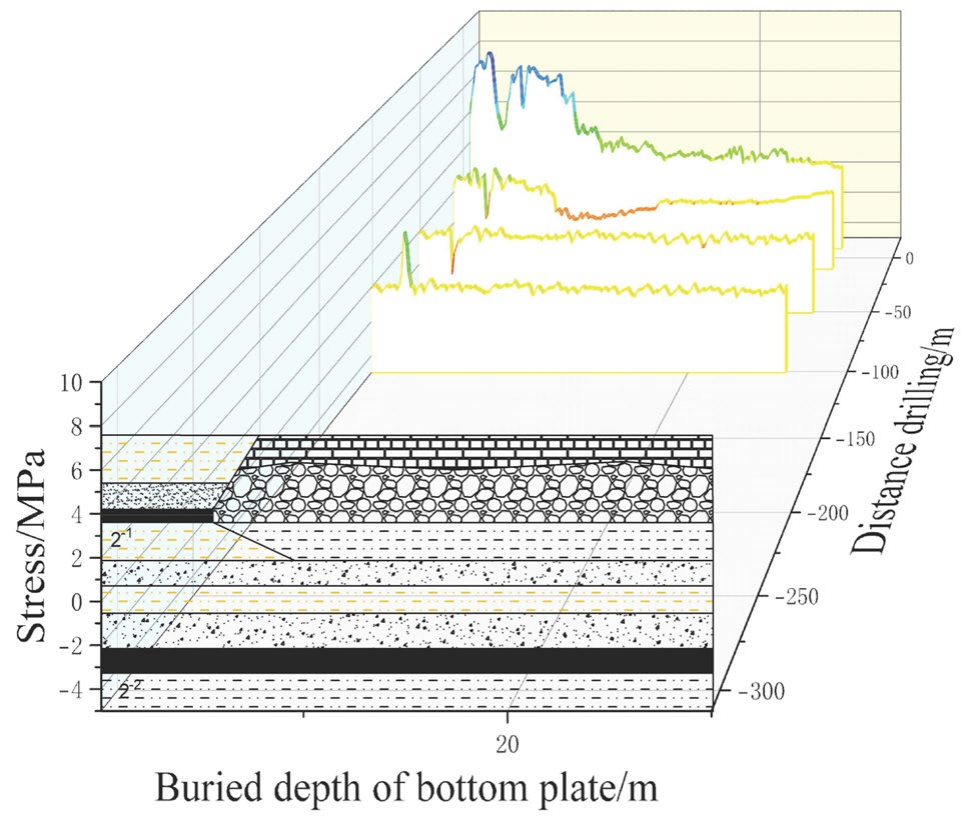
2# Borehole optical fiber monitoring data.

Figure 6 shows the stress variation curve obtained from the optical fiber cable in the 2# borehole, which is arranged along the same trend as the 1# borehole. The variation pattern is similar to that of the 1# borehole. From the Fig. 6, it can be observed that when the working face is 100 m away from the borehole, the sensing optical fiber cable inside the borehole is not influenced by mining activities, indicating a limited scope of advance influence from the working face. As the working face advances, when it is 50 m away from the borehole, the cable near the borehole entrance is the first to be affected, resulting in stress changes. This variation is mainly due to the concentrated stress on the floor strata caused by the coal pillar, where the pressure propagates downward, forming a shear stress plane and inducing strata deformation. However, the changes in the floor strata below the working face are within the range of error, indicating that the floor is still unaffected by mining activities at this point. When the working face advances to a distance of 10 m from the borehole, significant stress changes are measured in the cable below the working face inside the borehole. The strata at a burial depth of 8.15 m exhibit the highest stress variation, reaching 2.25 MPa, while the strata at a burial depth of 26.02 m show the smallest stress variation, only 0.06 MPa. At this stage, stress changes are mainly influenced by the concentrated stress from the working face, propagating downward. Shallow strata experience more significant stress concentration than deep strata. Once the working face advances beyond the 10 m mark, the floor strata are no longer affected by stress concentration. As the working face advances, the stress in the floor strata is released, resulting in a decrease in stress. The maximum stress release occurs at a burial depth of 6.22 m, with a strata stress variation of 6.33 MPa, while the minimum stress release occurs at a burial depth of 26.02 m, with a strata stress release of 0.22 MPa.

**Figure 7:**
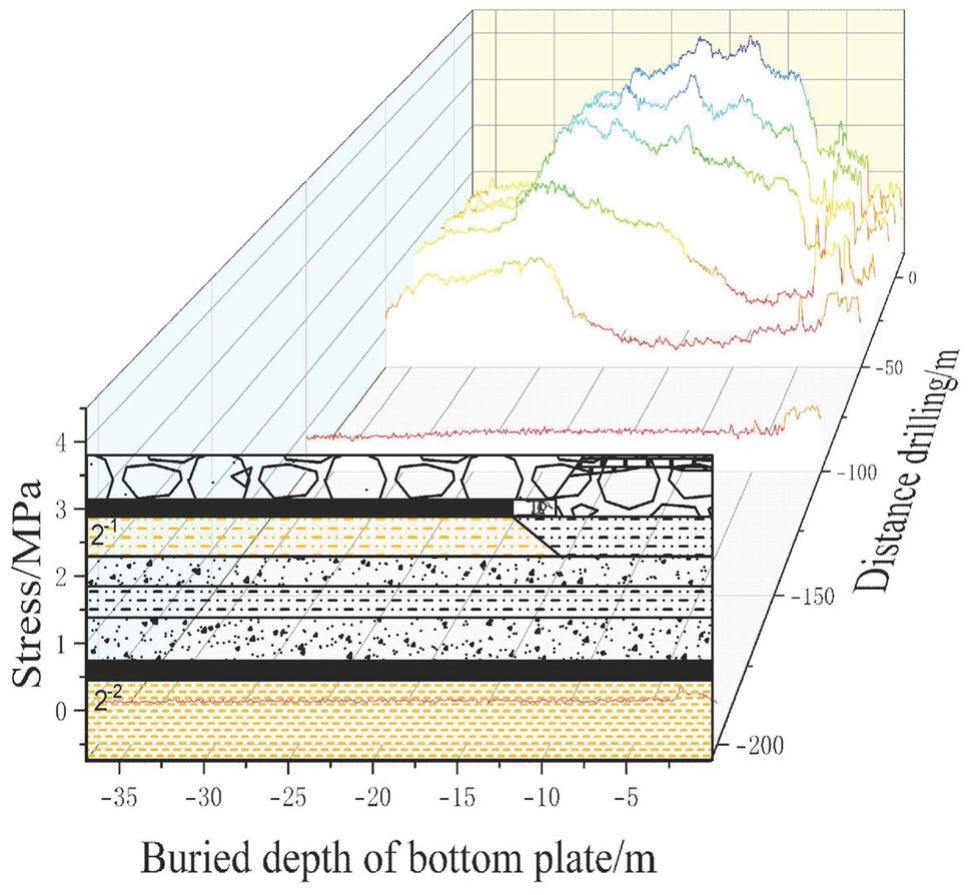
3# Drilling fiber monitoring data.

Figure 7 depicts the stress variation curve obtained from the optical fiber cable in the 3# borehole, which is arranged along the strike direction. The variation pattern differs slightly from that of the 1# and 2# boreholes. From the Fig. 7, it can be observed that when the working face is 200 m away from the borehole, stress changes occur in the cable at the borehole entrance and the 22 m section below it, influenced by the concentrated stress from the coal pillar and other factors. However, the sensing optical fiber cable below the working face inside the borehole is not affected by mining activities, indicating that the rock strata are still in their original stress state.

When the working face advances to a distance of 100 m from the borehole entrance, it is approximately 4.5 m horizontally above the bottom of the borehole. The rock layers located at the bottom of the borehole are influenced by the mining activities, leading to negative stress alterations in the rock layers from a depth of 25.87–36 m. Due to the greater depth of these rock layers, the stress changes are relatively small. The maximum stress concentration is observed at a depth of 33.75 m, with a stress change of 0.20 MPa, while the working face is situated 13.3 m away from the rock layers.

When the working face advances to a distance of 50 m from the borehole entrance, the rock layers above a depth of 20.12 m exhibit negative stress changes, indicating that this portion of the rock layers is influenced by stress concentration and is in the stress concentration stage. The maximum stress concentration occurs at a depth of 12.26 m, with a stress change of − 0.30 MPa. On the other hand, the rock layers below a depth of 20.12 m show positive stress changes, indicating that this portion of the rock layers is in the stress release stage. The maximum stress change in these layers is 1.40 MPa and occurs at a depth of 25.60 m.

When the working face advances to a distance of 30 m from the borehole entrance, the rock layers above a depth of 11.27 m exhibit negative stress changes, indicating that this portion of the rock layers is influenced by stress concentration and is in the stress concentration stage. The maximum stress concentration occurs at a depth of 12.26 m, with a stress change of − 0.27 MPa. On the other hand, the rock layers below a depth of 11.27 m show positive stress changes, and the stress changes continue to increase. This indicates that this portion of the rock layers is in the stress release stage. The maximum stress change in these layers is 2.19 MPa and occurs at a depth of 26.72 m.

When the working face advances to a distance of 100 m from the borehole, the working face has already passed beneath the rock layers below the borehole. Therefore, at this point, the fiber optic sensing in the borehole is not influenced by stress concentration. All the rock layers in the borehole experience an overall increase in stress during the upward migration phase, indicating that the entire rock mass has entered the stress release stage. The maximum stress of 2.91 MPa is reached at a depth of 25.88 m. As the working face continues to advance, the peak stress change in the sensing rock layers within the borehole will continue to migrate towards the shallower layers.

As the working face advances above the borehole, it has already passed through the rock layers within the sensing range of the borehole. The fiber optic cables in the shallow part of the borehole are located beneath the main haulage roadway of the working face and are influenced by roadway support measures. Therefore, during the monitoring period, the stress changes in these cables are relatively smaller compared to those below the working face. The fiber optic cables below the working face experience stress variations due to the stress release of the underlying strata. At this stage, the rock layer with the largest stress change is located at a depth of 16.89 m, while the working face has advanced 43.6 m beyond this layer. The stress change in this case is 3.53 MPa. The maximum stress change occurs in the middle section of the borehole, primarily because the stress release of the underlying strata is related to the distance the working face has advanced. At this point, the working face is closer to the shallow rock layers near the borehole, where the stress release of the rock mass is still increasing. This indicates that when the stress release of the rock mass reaches its maximum, the working face has advanced beyond 43.6 m. The rock layer with the minimum stress change is located at the bottom of the borehole, with a stress change of 0.50 MPa. At this stage, the stress in the entire sensing range of the borehole is increasing, indicating that the rock layers within the entire sensing range are in the stress release stage.

When the working face advances 10 m beyond the borehole, the rock layer with the maximum stress change has shifted to a depth of 10.69 m. At this point, the working face has advanced 43.27 m beyond this layer, resulting in a stress change of 4.08 MPa. The rock layer with the minimum stress change is located at the bottom of the borehole, with a reduced stress change of 0.47 MPa. This indicates that the rock layer at the bottom of the borehole has entered the stress recovery stage. The working face has already advanced 106 m beyond the borehole, entering the goaf. Within the entire borehole range, the fiber optic cables below a depth of 30.94 m exhibit relatively lower stress changes compared to earlier stages. This suggests that the rock layers below a depth of 30.94 m have entered the stress recovery stage.

Based on the above analysis, during the early stage of tunneling, only the rock layers beneath the roadway are affected by mining activities, resulting in increased stress changes in the floor rock layers primarily due to floor heave. The stress characteristics of the floor rock layers beneath the working face can be divided into four stages.

In the first stage, the original rock stress state is maintained. At this stage, the working face is located far from the rock layers, and mining activities have no impact on them, resulting in no stress changes.

The second stage is the stress concentration stage. As the working face advances, the advance support pressure leads to stress concentration in the floor rock layers. The stress concentration phenomenon increases and then decreases as the working face progresses. Once the rock layers return to their original stress state, the stress concentration stage ends.

The third stage is the stress release stage. When the working face passes through the rock layers, the stress in the floor rock layers is released.

The fourth stage is the stress recovery stage. As the working face continues to advance, the goaf gradually compacts, and the rock layers experience a peak stress followed by a gradual recovery. Eventually, the stress stabilizes and remains in a stable state.

In summary, the stress changes in the floor rock layers beneath the working face during tunneling can be categorized into four stages: the original stress state, stress concentration, stress release, and stress recovery.

### Vertical variation of stress information entropy of floor

**Figure 8:**
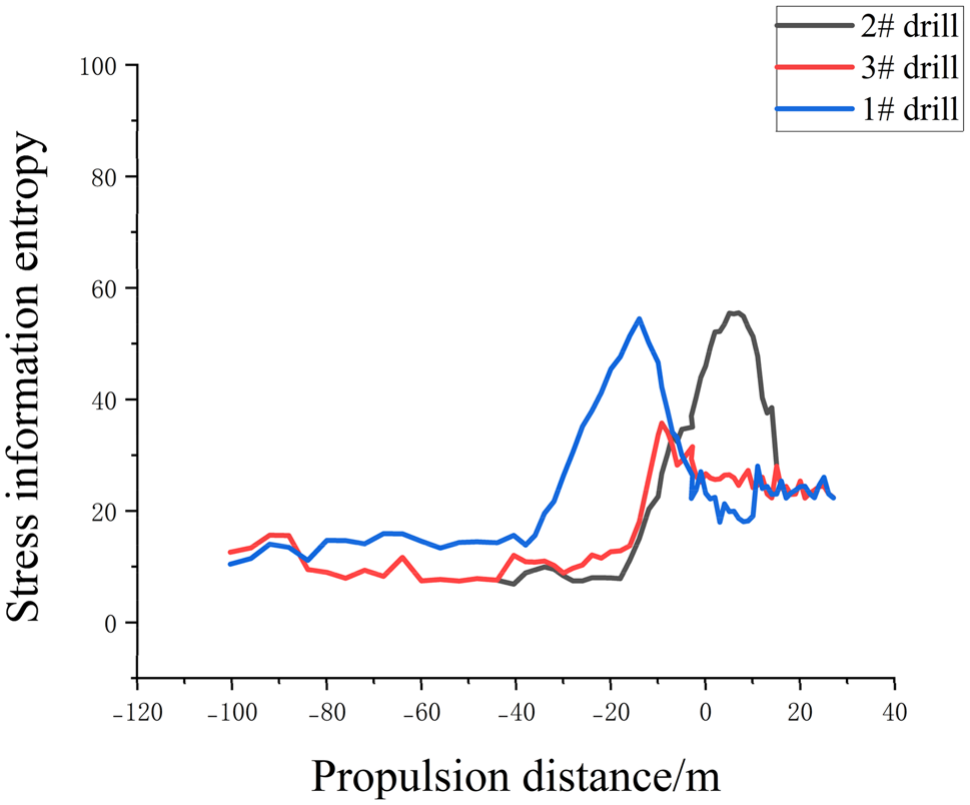
Schematic diagram of rock stress information entropy distribution during optical fiber monitoring of three boreholes.

To expand the ‘linear’ data obtained during each period of the working face advancement into ‘planar’ data along the boreholes, we complemented the data collected from three boreholes. The stress variations in the monitored rock layers were comprehensively analyzed, taking into account their depths and the advancement progress of the working face. Combining the stress changes observed through fiber optic monitoring during the working face advancement with the depth of the rock layers, we analyzed the variations in floor stress at different depths and advancement progress of the working face. This analysis resulted in the stress distribution profiles within the monitoring range of the three boreholes. By integrating the data and analysis from the three boreholes, we converted the linear data collected at different periods during the advancement of the working face into planar data along the boreholes. This comprehensive analysis examined the stress variations in the monitored rock layers and their correlation with the depth of the layers and the progress of the working face. By considering the depths of different rock layers, the advancement progress of the working face, and the changes in floor stress, we depicted the stress distribution within the monitoring range of the three boreholes in Fig. 8.

Figure 8 presents the distributed fiber optic monitoring results from the three boreholes during the advancement process. The graph illustrates the working face advancement distance ranging from − 120 to 40 m. Calculations were performed for the monitoring points at a rock layer depth of 5 m. Borehole 1 is inclined and located 13.72 m away from the working face coal pillar, positioned below the working face. Borehole 2 is inclined and located 5 m away from the working face coal pillar, situated beneath the main haulage roadway. Borehole 3 is inclined and located 2.82 m away from the working face coal pillar, also positioned beneath the main haulage roadway.

Observing Fig. 8, we can identify three stages as the mining distance increases: the original rock stress stage, the stress concentration stage, and the stress release stage. The entropy of stress information shows fluctuation, indicating the disorderliness in the overlying strata system caused by coal seam mining. The stress entropy experiences intense fluctuations, followed by growth and further increase. Throughout the monitoring period, the entropy of stress variation in the borehole-monitored rock layers displays significant fluctuations.

Borehole 1 shows pronounced fluctuations during the working face advancement from − 40 to − 0 m, while Boreholes 2 and 3 experience significant fluctuations from − 20 to 20 m. The variation is mainly attributed to different inclination positions and depths of the boreholes. The stress variations in the rock layers exhibit distinct stratification patterns concerning the advancement distance of the working face and the depth of the floor. Horizontally, the varying stress stages of the rock layers contribute to this phenomenon due to the different distances between the working face and the boreholes. Vertically, the stress variations show larger magnitude in the shallow layers and smaller magnitude in the deeper layers. This is because stress changes in the floor strata are influenced by the overlying coal-rock mass, transmitting stress changes from top to bottom. As a result, shallow layers experience larger stress variations while deeper layers exhibit smaller ones.

After 20 m, the entropy values for all three boreholes stabilize. With mining progress, the fractured rock layers of the working face undergo compaction, leading to decreased entropy values.

In summary, the analysis of the three borehole data sets, as shown in Fig. 8, provides comprehensive insights into the stress variations of the monitored rock layers during the working face advancement. The results highlight the stratified nature of stress changes, influenced by the distance between the working face and the boreholes as well as the depth of the floor. The observed fluctuations in stress entropy values indicate the dynamic behavior of the mining-induced stress variations.

### Information entropy change of one point stress in floor rock

In each of the three boreholes, 10 fiber optic gratings (FBGs) were implanted. The FBGs in Borehole 1 were named FBG1-01 to FBG1-10, in ascending order of their respective depths in the rock layers. Similarly, the FBGs in Borehole 2 were named FBG2-01 to FBG2-10, and the FBGs in Borehole 3 were named FBG3-01 to FBG3-10, all according to the depths of the rock layers they sensed.

This arrangement ensures a clear and logical identification of the fiber optic gratings in each borehole, minimizing repetition and facilitating efficient data analysis.

(1) 1# Drilled fiber Bragg grating

**Figure 9:**
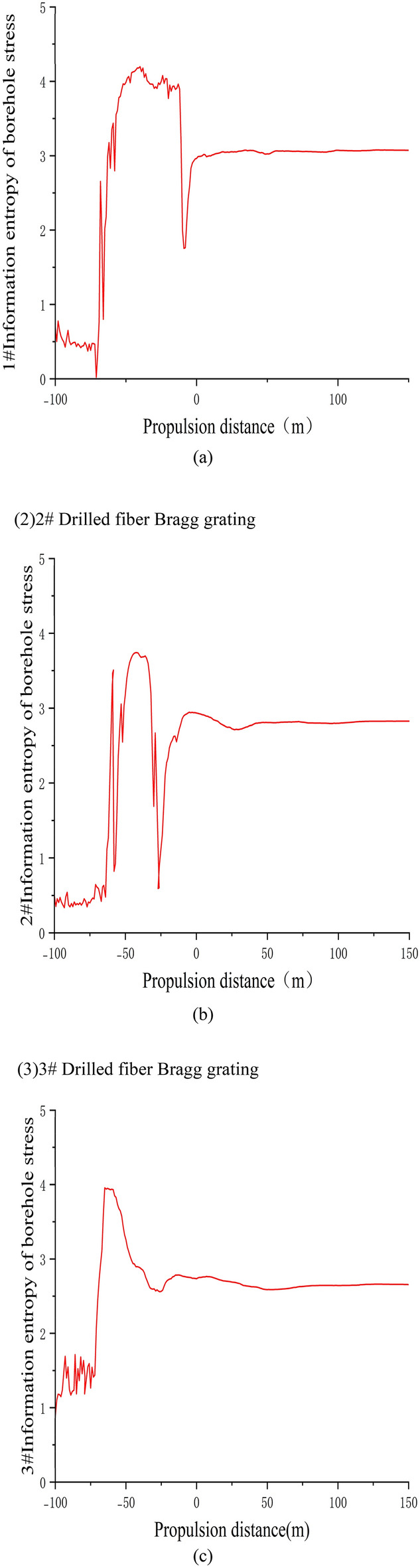
1#, 2#, 3# Borehole FBG stress information entropy.

Figure 9a depicts the variation curve of the stress information entropy in the sensing rock layers obtained by FBG1 in Borehole 1. The depths and distances of the FBG1 gratings from the coal pillar at the working face are as follows: FBG1-01 at a depth of 6 m and a distance of 17.40 m, FBG1-02 at a depth of 9.5 m and a distance of 30.45 m, FBG1-03 at a depth of 13 m and a distance of 43.50 m, FBG1-04 at a depth of 16.5 m and a distance of 56.60 m, FBG1-05 at a depth of 20 m and a distance of 69.64 m, FBG1-06 at a depth of 23.5 m and a distance of 82.70 m, FBG1-07 at a depth of 27 m and a distance of 95.75 m, FBG1-08 at a depth of 30.5 m and a distance of 108.85 m, FBG1-09 at a depth of 34 m and a distance of 121.90 m.

During the initial monitoring period, the working face was far from the sensors, and the floor strata were in their original stress state. As the working face advanced, the tunnel experienced floor heaving due to mining activities, resulting in the release of stress in the rock layers and an increase in stress variation. Furthermore, as the working face continued to advance, the stress variation steadily increased. In the range of − 100 to − 50 m of advancement, the entropy value exhibited a transition from slow fluctuations to significant growth. From − 50 to − 3 m, the entropy value tended to fluctuate mildly. Within the 0 m range, the entropy value initially experienced a drastic decline followed by an increase. Once the working face advanced beyond 0 m, the entropy value of the rock layers began to stabilize. This phenomenon can be attributed to the diminishing effect of stress concentration as the working face approached the sensors. The rock layers gradually returned to their original stress state, entering the stress release phase.

(2) 2# Drilled fiber Bragg grating

During the process of implanting the 10 fiber optic gratings in Borehole 2, FBG2-04 and FBG2-06 were damaged and unable to measure data. Figure 9b illustrates the stress variation curve of the sensing rock layers obtained by FBG2 in Borehole 2. The depths and distances of the FBG2 gratings from the coal pillar at the working face are as follows: FBG2-01 at a depth of 2.35 m and a distance of 2.35 m, FBG2-02 at a depth of 4.95 m and a distance of 4.95 m, FBG2-03 at a depth of 7.55 m and a distance of 7.55 m, FBG2-05 at a depth of 12.75 m and a distance of 12.75 m, and FBG2-07 at a depth of 17.95 m and a distance of 17.95 m.

During the initial monitoring period, the working face was far from the sensors, and the floor strata were in their original stress state. The entropy value exhibited a slow fluctuation and upward trend. When the working face advanced to − 64 m, the entropy value began to show drastic fluctuations and increased. In this stage, stress in the rock layers was released, and the variation in stress information entropy continued to increase. Subsequently, due to the compaction effect of the collapsed goaf in the roof strata, the change in stress entropy in the rock layers started to decrease. The rock layers entered the stress recovery region, and the variation in rock layer stress decreased. As the working face advanced within the 0 m range, the effect of stress concentration from the working face gradually diminished. When the working face reached the sensors, the rock layers returned to their original stress state, entering the stress release phase. The stress on the rock layers, measured by the gratings, began to be influenced by the advanced support pressure, and the rock layers entered the stress concentration phase. The variation in stress information entropy decreased, and the rock layers entered the stress recovery region. As the working face continued to advance, the variation in stress entropy of the rock layers started to stabilize.

(3) 3# Drilled fiber Bragg grating

The stress variation curves of the sensing rock layers measured by the 10 fiber optic gratings in Borehole 3 during the advancement of the working face are shown in Fig. 9c.

Figure 9c shows the change curve of stress information entropy of the sensing rock layer measured by the FBG3 grating in borehole 3. The FBG3-01 grating is buried at a depth of 4.5 m, with a dip position 2.32 m away from the coal pillar of the working face, a strike position 11.7 m away from the hole mouth, an FBG3-02 grating buried at a depth of 8 m, with a dip position 5.8 m away from the coal pillar of the working face, and a strike position 20.7 m away from the hole mouth; The FBG3-03 grating is buried at a depth of 11.5 m, with a dip position of 9.3 m from the coal pillar of the working face and a strike position of 29.7 m from the hole opening; The FBG3-04 grating is buried at a depth of 15 m, with a dip position of 12.80 m from the coal pillar of the working face and a strike position of 38.73 m from the hole opening; The FBG3-05 grating is buried at a depth of 18.5 m, with a dip position 16.3 m away from the coal pillar on the working face, and a strike position 47.77 m away from the orifice. The FBG3-06 grating is buried at a depth of 22 m, with a dip position 19.81 m away from the coal pillar of the working face and a strike position 56.80 m away from the hole opening; The FBG3-07 grating is buried at a depth of 25.5 m, with a dip position 23.3 m away from the coal pillar of the working face and a strike position 65.85 m away from the hole opening; FBG3-08 grating is buried at a depth of 29 m, with a dip position 26.81 m away from the coal pillar of the working face and a strike position 74.87 m away from the hole opening; The FBG3-09 grating is buried at a depth of 32.5 m, with a dip position of 30.3 m from the coal pillar of the working face and a strike position of 83.92 m from the hole opening; The FBG3-10 grating is buried at a depth of 36 m, with a dip position 33.8 m away from the coal pillar of the working face and a strike position 92.96 m away from the orifice. From the graph, it can be seen that as the working face advances, the roadway undergoes floor heave due to the influence of mining, and the stress in the rock layer is released, with a continuous increase in stress variation. When the working face advances to − 63 m, the entropy value reaches a peak of 3.94878. Later, due to the compaction of the goaf caused by the collapse of the roof rock layer, the change in stress entropy value of the rock layer begins to decrease. The rock layer enters the stress recovery zone, and the change in stress entropy of the rock layer decreases. Subsequently, it begins to be affected by the advanced support pressure. The rock layer enters the stress concentration stage, and the change in stress information entropy begins to decrease. When the working face pushes past the sensor, the rock layer returns to its original stress state, The rock strata enter the stress release stage, and as the working face continues to advance, the change in stress entropy of the rock strata decreases, and the change in stress information entropy of the rock strata begins to stabilize.

Based on the stress variation patterns of the three boreholes, it can be seen that the stress variation follows the advancing distance of the working face, presenting four stages of “original rock stress—stress concentration—stress release—stress recovery”. Within these four stages, the changes in stress information entropy of the rock layers are not the same.

During the original rock stress stage, the mining of the working face still has an impact, and the stress entropy value of the rock layer at the bottom of the working face has little change.

During the stress concentration stage, the change in stress information entropy of rock layers begins to exhibit drastic fluctuations in price. This stage of stress change is caused by advanced stress concentration in the working face. The stress in the non-collapsed rock layer above the goaf is concentrated in front of the working face, and the stress is transmitted from top to bottom. The shallow rock layer is greatly affected by stress concentration, while the deep rock layer is less affected by stress concentration.

During the stress release stage, due to the pushing of the working face and the release of stress in the goaf, the rock stress undergoes a drastic change, and the amount of change in the information entropy of rock stress continues to increase. The changes in floor stress are all caused by the mining of the upper protective layer. After the mining of the protective layer, the stress in the goaf is released, and the stress is transmitted from top to bottom. During the stress transmission process, the stress caused by the expansion and deformation of the rock layer is consumed layer by layer until the end. Therefore, the stress in the shallow floor rock layer increases, while the stress entropy changes in the deep floor rock layer decreases.

In the stress release stage, after the change in stress entropy of the rock layer reaches its peak, the amount of stress change in the rock layer gradually decreases. The initial stress change is relatively intense, and then gradually stabilizes as the working face advances. In the stress recovery stage, the floor rock layer of the goaf collapses, causing the floor rock layer to gradually compact. The stress of the rock layer begins to recover, and the collapsed roof rock layer directly acts on the floor of the 2–1 coal seam, and the shallower buried rock layer is first compacted by the collapsed roof. During the compaction process, the stress is consumed again, and the stress transmitted to the deep rock layer decreases layer by layer until there is no impact. The stress change of the deeper buried rock layer is relatively small. During the stress release stage of the floor rock layer, the stress in the rock layer undergoes drastic changes. From the stress change curve of the rock layer, it can be seen that the stress change in the shallow buried rock layer presents a stepped shape, while the stress change in the deeper buried rock layer is relatively smooth compared to the curve. This is precisely due to the stress transmission characteristics of the bottom plate. Shallow buried rock layers are directly affected by goaf mining, and stress changes are basically only affected by goaf stress changes. Therefore, the stress changes of the rock layers are consistent with the advancing of the working face and the collapse state of the roof rock layers, that is, the stress changes of the rock layers are more sensitive to the stress changes of the goaf. However, due to the stress loss during the stress transmission process, the stress transmission in the goaf of deep rock layers is related to the expansion/compaction state of the upper rock layer and the magnitude of the transmitted stress. Multiple influencing factors during the stress transmission process cause the stress changes in the bottom rock layer to be less sensitive to the stress changes in the goaf.

In the context of coal and gas outburst prevention during protective layer mining, the reduction of stress within the coal-rock strata and the opening of innate pores and fissures enhances the permeability of the protective layer. This process leads to a decrease in gas pressure within the coal seam, achieving a decompression effect. As for the prevention of rock burst, even a minimal reduction in the stress of the protective layer can still contain a substantial quantity of elastic energy internally. It is only when the stress is diminished to a specific extent that an effective reduction can be realized and rock burst can be prevented. Consequently, accurately determining the critical rate of stress release is of utmost importance for assessing the effectiveness of decompression. This paper discusses the mining of the protective layer in the Hulu Su Mine, analyzing the patterns of floor failure within the protective layer, and identifies the decompression range of the 2–2 coal seam therein. These insights provide theoretical support for the staggered arrangement of the 2–2 working face and the upper protective layer, ensuring effective pressure reduction and the safe and efficient retrieval of the working face.

With the alleviation of information entropy in the stress of coal and rock layers during the prevention of coal and gas outburst in protective layer mining, an increase in permeability of the protective layer is often accompanied by the activation of original pore fractures. As a result, the gas pressure within the coal seam is reduced, achieving the desired decompression effect. In terms of rock burst prevention, even a slight decrease in the stress information entropy of the protective layer might leave behind a large amount of elastic energy. Only when the information entropy of the stress has been sufficiently reduced, can decompression be effectively achieved, and the incidence of rock burst be prevented. Therefore, in subsequent research, the evaluation of decompression effects can be improved through the reasonable determination of the critical release rate of stress information entropy.

## On site monitoring effect verification of floor coal and rock mass

### Numerical simulation

**Figure 10:**
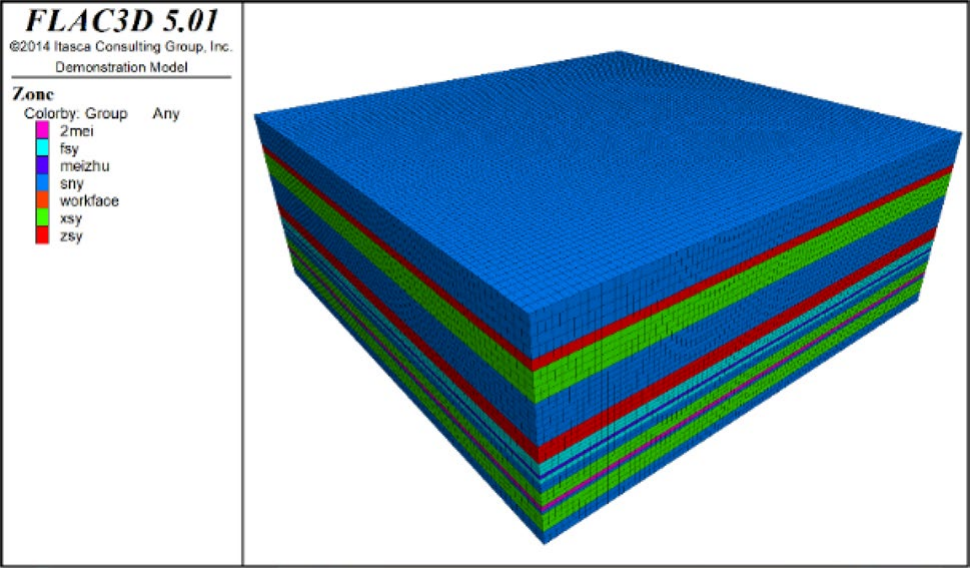
Numerical simulation calculation model.

Based on the geological conditions and rock physical–mechanical parameters of the Hulusu Coal Mine's 21104 working face, a numerical simulation model was established. In this model, the simulated length of the 21104 working face is 320 m in the direction of advance, and the simulated height is 112 m. The stress loading method was employed to simulate the unmodeled strata, and the coal seams had a dip angle of 0°. The specific model diagram is shown in Fig. 10.

In the Flac3D numerical simulation analysis, it was discerned that following the extraction of coal seams via the direct excavation method, there is an absence of stress contact calculation between the roof and floor strata. When examining the stress distribution pattern of the floor, one can unequivocally observe that the stress within the floor is in a state of complete decompression, which contradicts actual conditions. Therefore, when simulating and analyzing the stress and decompression patterns of protective layer exploitation and caving, it is imperative to execute contact calculations for the void space’s roof and floor.

To authentically simulate the stress transfer from the overlying collapsed rock mass to the floor following coal seam recovery, the material’s constitutive model, employed both for initial equilibrium and excavation computations, is based on the Mohr–Coulomb criterion—a principle grounded in elasticity and plastic flow theories, constructing a correlation between strain and stress, adeptly delineating the transformation between elastic and plastic phases in rock mass materials. Upon reaching equilibrium post-excavation, the collapsed debris within the void space is treated with backfill. The chosen constitutive model for the fill material is the Double-Yield criterion, which takes into account shearing and associated modulus coefficients, thus accurately reflecting the characteristic behavior of the collapsed rock body.

Subsequently, a rudimentary block of dimensions 1 m × 1 m × 1 m is constructed. By altering mechanical parameters such as shear modulus, bulk modulus, and cohesion, and by simulating the rock’s uniaxial compressive strength, the outputs are calibrated to align with the stress–strain relationship for fractured rock bodies as proposed by SALAMONI, delineated in Eq. (4). The process culminates in the post-filling equilibrium calculation, effectively facilitating the transfer of stress between the roof and floor strata of the overlying collapsed rock mass.

4 $$\sigma = \frac{{10.39\varepsilon \sigma_{{\text{c}}}^{1.032} (1 - {\text{b}})}}{{{\text{b}}^{7.7} (1 - {\text{b}} + {\text{b}}\varepsilon )}}$$

Within the formula, ${\sigma }_{c}$ denotes the uniaxial compressive strength; b represents the bulking factor; $\sigma$ stands for the vertical stress endured by the collapsed rock mass; and $\varepsilon$ is the strain of the collapsed rock mass.

To ensure the elimination of boundary effects in the computational results, the model was designed with coal pillars of 80 m width on the front, back, left, and right sides of the working face. The working face advanced 20 m during each mining cycle, totaling 16 cycles and a cumulative distance of 320 m. Horizontal displacements of u = ± 0.01 m were imposed on the four surfaces of the model, while a vertical displacement of w = ± 0.01 m was applied to the model's bottom surface. The top surface of the model was considered as a free boundary. A vertical stress of 10.6 MPa was applied to the top surface of the model in a downward direction. The numerical simulation adopted the Mohr–Coulomb constitutive model, and the gravitational acceleration was set at 9.8 m/s^2^ in the vertical downward direction.

**Figure 11:**
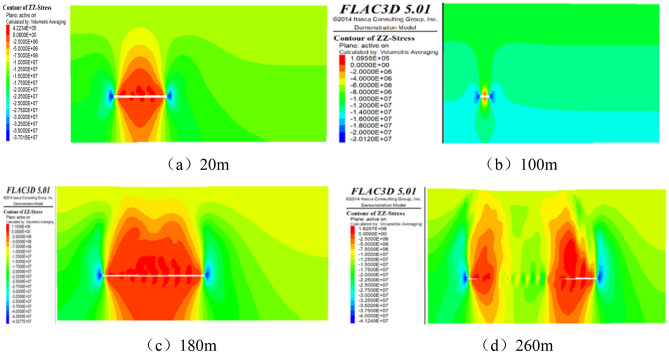
Stress nephogram during advancing of working face.

By using FLAC3D software to mine the upper protective layer 2–1 coal seam, the mining direction is from left to right. The vertical stress cloud maps of the working face advancing to 20 m, 100 m, 180 m, and 260 m are captured, and the results are as follows. The results are shown in Fig. 11.

We can know from Fig. 11, when the mining face advances to 20 m, the movement of the working face disrupts the equilibrium of the original rock stress, leading to stress redistribution. The vertical stress of the working face exhibits a clear zoning phenomenon in both the horizontal and vertical directions.

In the horizontal direction, the vertical stress of the rock mass outside a 25 m range in front of the coal wall of the working face is 15.7 MPa, which is almost consistent with the actual vertical stress of 16.0 MPa at that depth. Therefore, this area belongs to the original rock stress zone. Within a 20 m range in front of the coal wall, the vertical stress of the coal-rock mass is higher than the original rock stress, reaching a maximum of 20.12 MPa. In the goaf area behind the coal pillar within a 25 m range, the rock mass is subjected to concentrated mining pressure, indicating a concentrated pressure zone with a maximum stress of 20.12 MPa. The floor of the goaf is in a stress reduction zone since the working face has not advanced significantly, and the overlying strata have not collapsed. Therefore, the stress acting on the floor is relatively small.

In the vertical direction, certain areas of the floor experience an upward vertical stress of 0.09 MPa, indicating the occurrence of floor heaving. As the depth of the rock mass increases, the stress on the floor of the goaf gradually increases and changes to a vertical downward direction. The stress distribution exhibits a "U" shape, with stress gradient values of 2.5 MPa, 5.0 MPa, 7.5 MPa, and 10.0 MPa, respectively. The vertical stress value beneath the floor of the goaf is 16.5 MPa at a depth of 60 m, which is comparable to the original rock stress value of 16.7 MPa at the same depth. Beyond the 60 m range beneath the floor, it belongs to the original rock stress zone after the extraction of the protective layer 2–1 coal seam.

When the mining face advances to 100 m, the concentration of mining pressure becomes more pronounced, primarily in front of the coal wall of the working face and behind the coal wall in the goaf area.

In the horizontal direction, the maximum mining pressure at the coal wall of the working face is 37 MPa, which is an increase of 224.2% compared to the vertical stress at that burial depth. The vertical stress near the floor in proximity to the working face and goaf area is 4.22 MPa, indicating an upward stress direction and floor expansion deformation. The vertical stress in the middle part of the goaf floor is 2.5 MPa, indicating a downward stress direction. This implies that the sides of the floor are more susceptible to lower stress and more likely to experience floor heaving compared to the middle section. The average vertical stress of the floor is approximately 2 MPa, accounting for 12.7% of the original rock stress value. The coal wall behind the goaf area is subjected to concentrated mining pressure, with the stress value increasing from 20.1 to 37 MPa.

In the vertical direction, as the depth of the floor increases, the vertical stress gradient of the floor is 1.64 MPa, 2.5 MPa, and 5 MPa, respectively.

When the mining face advances to 180 m, the mining pressure in front of the coal wall of the working face and behind the goaf area continues to increase.

In the horizontal direction, the peak stress at the working face is 43.27 MPa, which is an increase of 262.24% compared to the vertical stress of the original rock. The minimum vertical stress at the floor position of the working face is 1.15 MPa, indicating a decrease in stress values in the goaf floor as the distance of the working face advances.

In the vertical direction, the width of the "U"-shaped distribution of the floor's vertical stress increases, and its influence area expands with the advancement of the working face. The vertical stress on the floor decreases to only 1.15 MPa, indicating a gradual reduction in the lateral support pressure on the floor as the distance of the working face increases.

When the mining face advances to 260 m, in the horizontal direction, the stress peak in front of the coal wall of the working face is 41.2 MPa, which is essentially the same as the peak stress at 180 m. This suggests that the stress concentration phenomenon caused by the overlying strata on the coal wall has reached its peak and will not increase further with the advancement of the working face.

In the vertical direction, due to the compaction caused by the collapse of the overlying strata, the rock layers in the middle of the goaf have essentially returned to their original stress state. The number of rectangular stress bands on the floor has increased, and the vertical stress of the floor layers unaffected by the compaction of the floor has increased to 1.82 MPa. This phenomenon is mainly attributed to the collapse of the goaf roof.

When the mining face advances to 320 m, in the horizontal direction, the stress peak in front of the coal wall of the working face stabilizes at 41.2 MPa. In the vertical direction, the number of rectangular stress bands on the floor increases, and the vertical stress remains constant at 2.1 MPa.

Vertically, as the working face continues to advance, the range and depth affected by stress release on the floor further increase.

**Figure 12:**
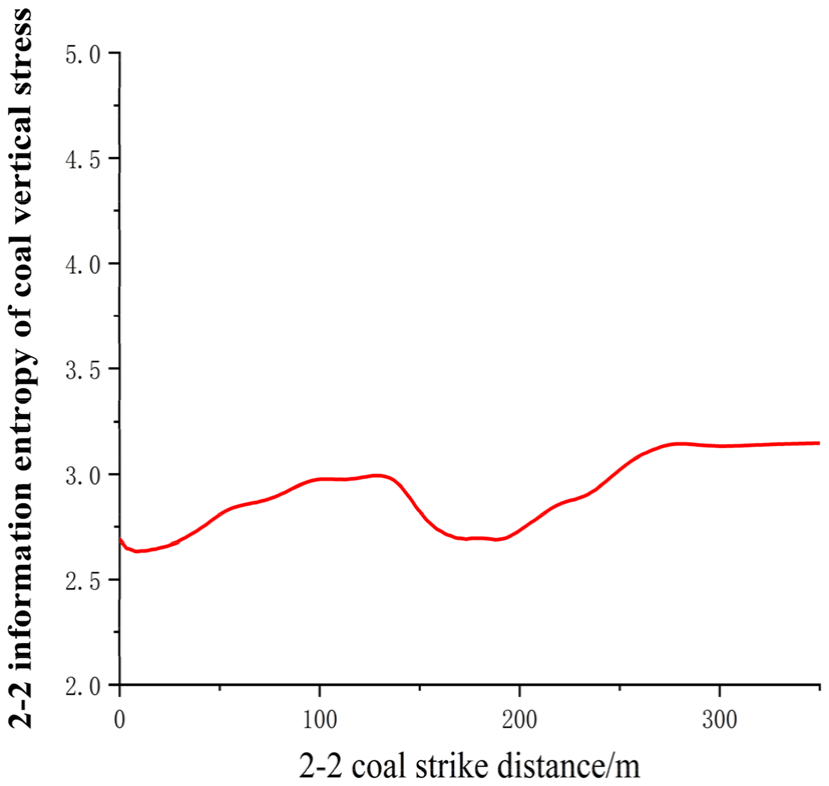
2–2 Vertical stress distribution of coal.

In conclusion, during the extraction process of the overlying protective layer, the vertical stress distribution on the floor exhibits an evolution from "U"-shaped to "v"-shaped to rectangular stress bands. The changes in floor layer stress are greatly influenced by the collapse of the goaf roof. When the rock layers in the goaf collapse, the variation of vertical stress entropy in the coal layer of the working face is measured using vertical stress measuring lines with a spacing of 10 m in the numerical simulation experiment on face 22104. The results are shown in Fig. 12.

According to Fig. 12, it can be observed that the coal body of the 21104-working face, located behind the goaf and below the coal wall of the working face, is subjected to concentrated mining pressure, resulting in significant vertical stress. The vertical stress entropy of the coal body beneath the goaf area, corresponding to the 22104-working face, is generally less than 2.75.

As the upper protective layer of the 21104 working face advances forward, the stress concentration phenomenon in front of the coal wall of the working face intensifies, leading to an increasing peak in stress entropy. In the goaf area behind the working face, the stress gradually recovers due to the recompaction of the collapsed zone rock layers. However, the stress entropy value in this area remains smaller than the peak stress entropy in front of the coal wall. The middle section of the goaf area experiences stress reduction, and this stress state propagates downward towards the floor. Therefore, the stress conditions of the coal body of the 22104-working face below the floor of the 21104-working face are essentially similar to those of the floor of the 21104-working face.

### Similarity simulation

The experiment focused on the stress characteristics of the coal-rock mass in the 21104 working face and its floor at the Hulusu coal mine. The mining geological conditions of the working face were taken as the prototype, and a plane stress model with dimensions of 3000 mm (length) × 200 mm (width) × 1350 mm (height) was constructed. In accordance with the principles of similarity theory, the model experiment needed to be geometrically, kinematically, and dynamically similar to the prototype system. Therefore, the geometric similarity ratio, bulk density similarity ratio, and other similar parameters were determined, as shown in Table 2.

**Table 2:** Model similarity constant.

Similar name	Parameter	Similar name	Parameter
Geometric similarity ratio	1:150	Bulk density similarity ratio	1:1.56
Displacement similarity ratio	1:150	Time similarity ratio	1:12.25
Stress similarity ratio	1:234	Speed similarity ratio	1:12.25
Strain similarity ratio	1:234	Similarity ratio of elastic modulus	1:234
Strength similarity ratio	1:234	Gravity acceleration similarity ratio	1:1

**Figure 13:**
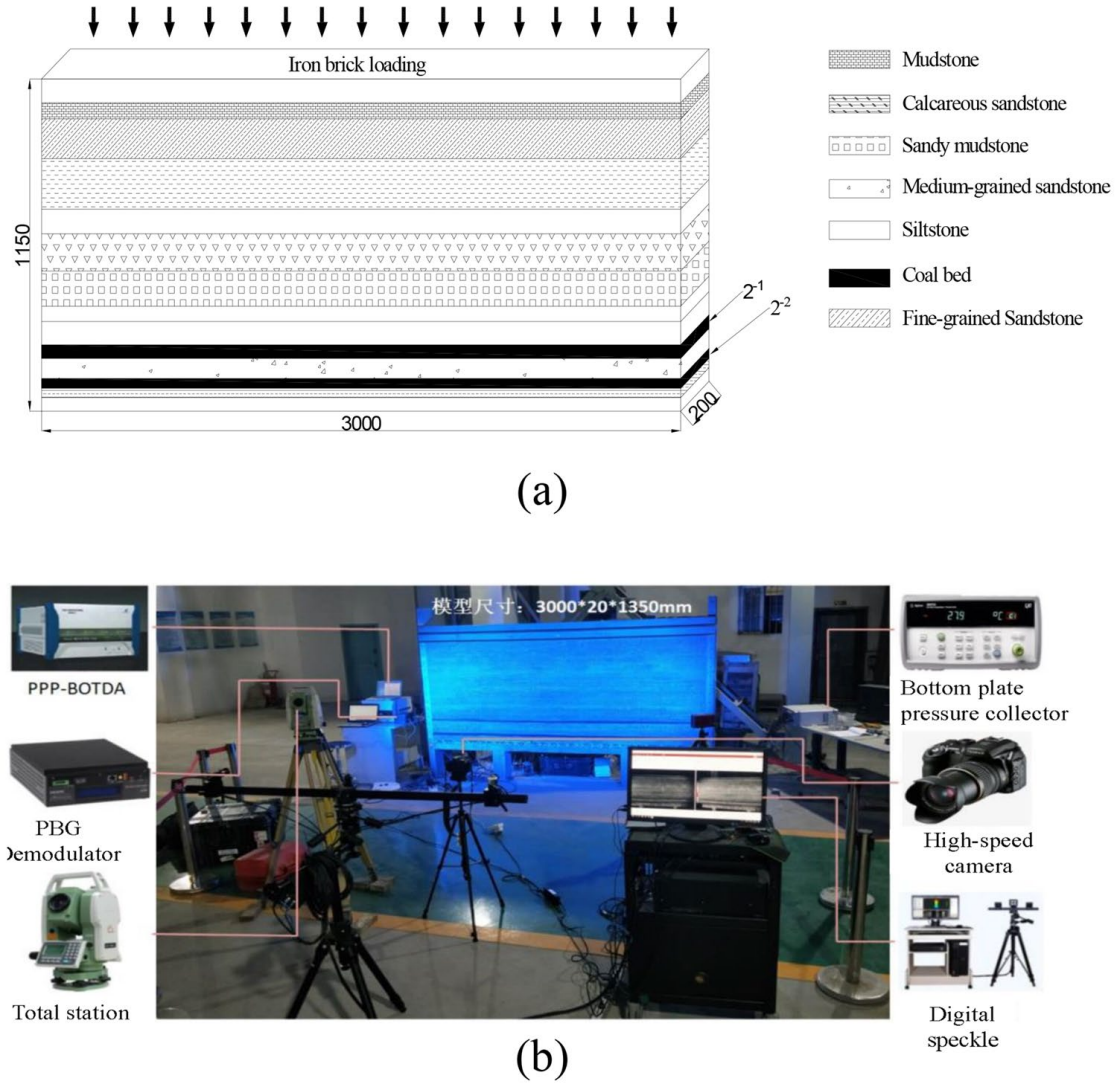
Schematic diagram of model.

In this experiment, river sand, fly ash, and clay were used as aggregates for the rock (coal) layers, while gypsum and calcium carbonate (chalk) were used as binding materials. Mica powder was used as a stratification material, and water was used as the mixing agent. Based on the physical and mechanical parameters of the prototype rock layers, appropriate mix ratios were selected^35^. Then, according to the determined stratigraphic sequence and mix ratios, various similar materials were thoroughly mixed, paved evenly, and compacted. The model was constructed layer by layer from bottom to top, as shown in Fig. 13.

**Figure 14:**
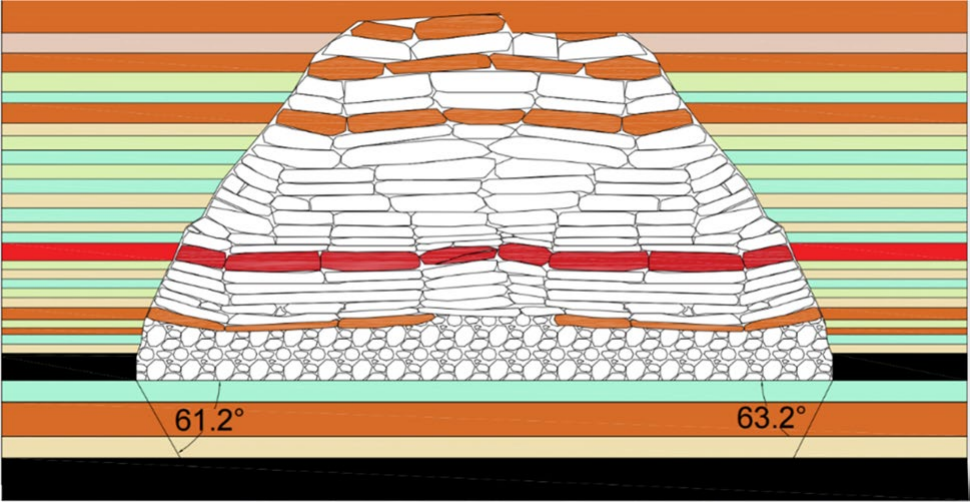
Schematic diagram of mining scope.

In the similarity model, boundary coal pillars of 300 mm were left on both sides. The total length of coal seam advancement was set at 2400 mm, with a mining slot width of 60 mm. Each cut advanced by 30 mm until the completion of mining. After each mining operation, detailed records and observations were made, and data were collected using the aforementioned monitoring system, until the completion of the working face excavation. The entire model experiment extensively observed and documented the complete process of stress variation in the underlying rock layers due to mining^36^. The mining scope is shown in Fig. 14.

In physical model 2–2, 60 pressure sensors were uniformly distributed on the coal floor, enabling real-time monitoring of the stress variation in the coal floor during the coal mining process in model 2–1. Before the excavation of the working face, the monitoring data from the pressure sensors on the floor were recorded as initial values. Subsequently, the stress variation values of the floor pressure sensors were recorded for each mining operation. The measured results from the floor pressure sensors were converted into prototype stress values based on the similarity ratio of stresses in the model.

**Figure 15:**
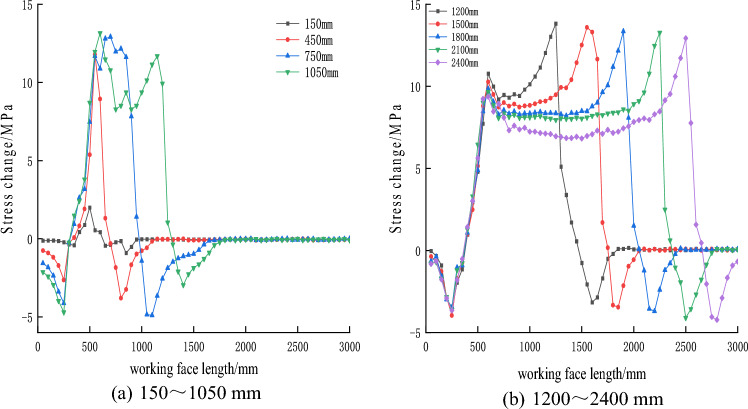
Variation law of floor stress at different distances of working face advance.

The distribution pattern of floor stress as the working face advanced along the strike is shown in Fig. 15. From the graph, it can be observed that as the working face advanced, the stress beneath the goaf gradually dissipated, resulting in an increasing stress variation value. The floor beneath the coal pillars on both sides of the goaf exhibited stress concentration, with decreasing stress variation values.

When the working face advanced by 150 mm, the stress beneath the goaf significantly reduced, while the stress on the sides of the goaf increased due to stress concentration. At 450 mm of working face advancement, the stress release in the floor beneath the goaf further increased, reaching a maximum of 12.27 MPa, and the stress concentration phenomenon in the floor on both sides of the goaf became more pronounced. When the working face continued to advance by 750 mm, the stress release in the floor beneath the goaf reached a maximum of 12.91 MPa, similar to the value at 450 mm of working face advancement. This indicates that the floor beneath the advancing working face was sufficiently affected by mining. At this point, the stress in some rock layers beneath the goaf recovered to a maximum value of 8.27 MPa, suggesting the development of a roof caving zone and gradual compaction of the goaf's debris^37^.

As the working face advanced from 1200 to 2400 mm, the peak stresses in the floor rock layers and the stress concentration peak in front of the working face remained relatively stable. The positions of the peak stresses in the floor rock layers, the stress concentration peak in front of the working face, and the recovery position of the floor rock layer stress all advanced with the progress of the working face and were generally consistent with the working face advancement distance. The amount of stress recovery in the floor rock layers decreased as the working face advanced and the caving zone of the roof rock layers increased. At 1200 mm of working face advancement, the stress variation in the floor rock layers was 9.5 MPa, and at 2400 mm, it reduced to 6.8 MPa, indicating a gradual approach of the floor rock layer stress to the original rock stress as the working face advanced.

**Figure 16:**
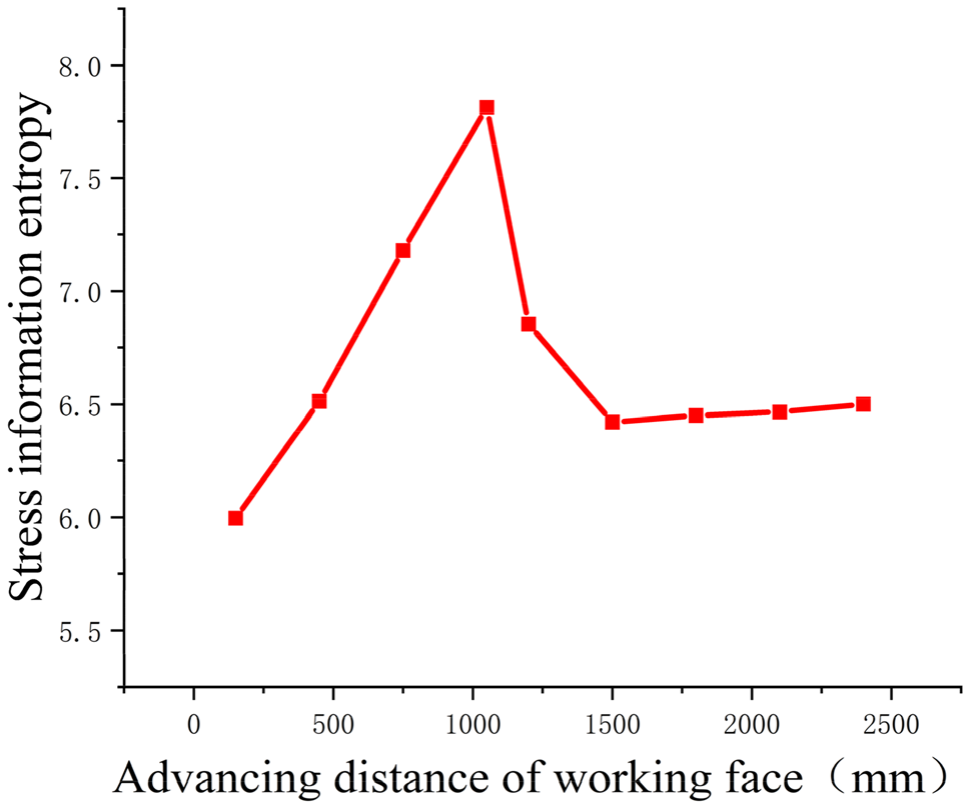
Variation law of stress information entropy of floor at different distances of working face.

The distribution pattern of floor stress along the advancement of the working face in the strike direction is shown in Fig. 16. From Fig. 16, it can be observed that as the working face advances, the stress beneath the goaf is released, resulting in an increase in stress entropy. The stress on the sides of the goaf increases due to stress concentration. At 450 mm of working face advancement, the stress release in the floor beneath the goaf further increases. When the working face continues to advance by 750 mm, the stress entropy in the floor beneath the goaf reaches a maximum value of 7.26783. At 1000 mm of working face advancement, the stress in the floor rock layers reaches its peak. As the working face advances to 1200 mm, the stress entropy in the floor rock layers starts to decrease. When the working face advances to 1500 mm, the stress variation in the floor rock layers decreases to 5.94694, indicating a recovery of the stress entropy in the floor rock layers towards the entropy value of the original rock stress.

From Fig. 16, it can be observed that the stress entropy undergoes an initial increase, followed by a decrease, and then a gradual and slight rise. This phenomenon is mainly attributed to the collapse of the roof rock layers at the cutting point, leading to partial stress recovery in the floor rock layers. The floor rock layers behind the working face on the right side have not collapsed yet, resulting in a stress release peak in the floor rock layers^38^. There is a smooth transition zone between the two peaks, which is caused by the compaction resulting from the collapse of the roof rock layers.

## Conclusion

1. By using Brillouin fiber optic sensing technology, real-time monitoring of stress changes and failure characteristics of the bottom rock mass during protective layer mining has been achieved, revealing the spatiotemporal evolution law of stress information entropy of the protected layer during upper protective layer mining. From the perspective of changes in stress information entropy, mining leads to a decrease in the orderliness of the entire overlying rock system and an increase in stress information entropy.
2. According to the evolution characteristics of stress information entropy, it is divided into four stages, namely the original rock stress stage, stress concentration stage, stress release stage, and stress recovery stage. The bottom plate is affected by advanced stress concentration starting from 30 to 70 m in front of the working face. The working face pushes past the bottom plate 8–35 m, and the bottom plate begins to relieve pressure. The maximum stress change is located 50–65 m behind the working face. The working face pushes past the bottom plate 130–150 m and enters the stable stage.
3. In different spatial evolution processes, there are significant differences in stress information entropy. In the horizontal direction, when the distance between the working face and the borehole is different, the stress change stage of the rock layer is different, resulting in different changes in stress information entropy. In the vertical direction, the entropy change of stress information in rock layers shows a large change in entropy value in shallow rock layers, while a small change in entropy value in deep rock layers.

## Data Availability

The data used to support the findings of this study are available from the corresponding author upon request.
